# Imbalances in circulating angiogenic factors in the pathophysiology of preeclampsia and related disorders

**DOI:** 10.1016/j.ajog.2020.10.022

**Published:** 2022-02

**Authors:** Sarosh Rana, Suzanne D. Burke, S. Ananth Karumanchi

**Affiliations:** a Division of Maternal-Fetal Medicine, Department of Obstetrics and Gynecology, The University of Chicago, Chicago, IL; b Department of Medicine, Obstetrics, and Gynecology, Beth Israel Deaconess Medical Center, Harvard Medical School, Boston, MA; c Department of Medicine, Cedars-Sinai Medical Center, Los Angeles, CA

**Keywords:** bronchopulmonary dysplasia, cardiovascular disease, fetal death, intrauterine growth restriction, fetal hydrops, glomerular endotheliosis, hypertension, placental-derived growth factor, proteinuria, soluble endoglin, spiral artery remodeling, twin-to-twin transfusion syndrome, vascular endothelial growth factor

## Abstract

Preeclampsia is a devastating medical complication of pregnancy that can lead to significant maternal and fetal morbidity and mortality. It is currently believed that there is abnormal placentation in as early as the first trimester in women destined to develop preeclampsia. Although the etiology of the abnormal placentation is being debated, numerous epidemiologic and experimental studies suggest that imbalances in circulating angiogenic factors released from the placenta are responsible for the maternal signs and symptoms of preeclampsia. In particular, circulating levels of soluble fms-like tyrosine kinase 1, an antiangiogenic factor, are markedly increased in women with preeclampsia, whereas free levels of its ligand, placental, growth factor are markedly diminished. Alterations in these angiogenic factors precede the onset of clinical signs of preeclampsia and correlate with disease severity. Recently, the availability of automated assays for the measurement of angiogenic biomarkers in the plasma, serum, and urine has helped investigators worldwide to demonstrate a key role for these factors in the clinical diagnosis and prediction of preeclampsia. Numerous studies have reported that circulating angiogenic biomarkers have a very high negative predictive value to rule out clinical disease among women with suspected preeclampsia. These blood-based biomarkers have provided a valuable tool to clinicians to accelerate the time to clinical diagnosis and minimize maternal adverse outcomes in women with preeclampsia. Angiogenic biomarkers have also been useful to elucidate the pathogenesis of related disorders of abnormal placentation such as intrauterine growth restriction, intrauterine fetal death, twin-to-twin transfusion syndrome, and fetal hydrops. In summary, the discovery and characterization of angiogenic proteins of placental origin have provided clinicians a noninvasive blood-based tool to monitor placental function and health and for early detection of disorders of placentation. Uncovering the mechanisms of altered angiogenic factors in preeclampsia and related disorders of placentation may provide insights into novel preventive and therapeutic options.

## Introduction

Preeclampsia (PE), a pregnancy-related disorder that affects 3% to 4% of pregnancies, is a major cause of maternal and fetal morbidity and mortality.^1^^,^^2^ Delivery of the placenta has been shown to resolve the acute clinical symptoms of PE, suggesting that the placenta plays a central role in the pathogenesis of PE.^3^^,^^4^ When it occurs after delivery perhaps owing to the presence of retained placental fragments, PE can be treated by uterine curettage.^5^^,^^6^ During normal pregnancy, the placental bed and uterine circulation undergo dramatic vascularization to enable circulation between the fetus and the mother. Placental vascularization involves vasculogenesis, angiogenesis, and maternal spiral artery remodeling.^10^, ^7^, ^8^, ^9^ These processes require a delicate balance of molecules that regulate angiogenesis and vessel remodeling. Defects in adequate vascularization and vascular remodeling lead to placental ischemia and injury.^11^^,^^12^ In women with PE, several antiangiogenic factors, such as soluble fms-like tyrosine kinase 1 (sFLT1) and soluble endoglin (sENG), are produced by abnormal placentas in higher than normal quantities and released into maternal circulation.^13^, ^14^, ^15^, ^16^, ^17^, ^18^ The imbalance of proangiogenic and antiangiogenic factors in circulation is thought to trigger the onset of PE by inducing microangiopathy in target organs such as the kidney, liver, or brain.^19^ The extent to which underlying genetics and environmental stressors contribute to the overproduction of antiangiogenic factors by the placenta in PE is still being debated. This review will focus on the normal vascular development in the placenta, the angiogenic imbalance that occurs during PE, and the role of angiogenic biomarkers in the clinical diagnosis and prediction of PE and related placentation disorders.

## Placental Vascular Development in Health and in Disease

**Figure 1:**
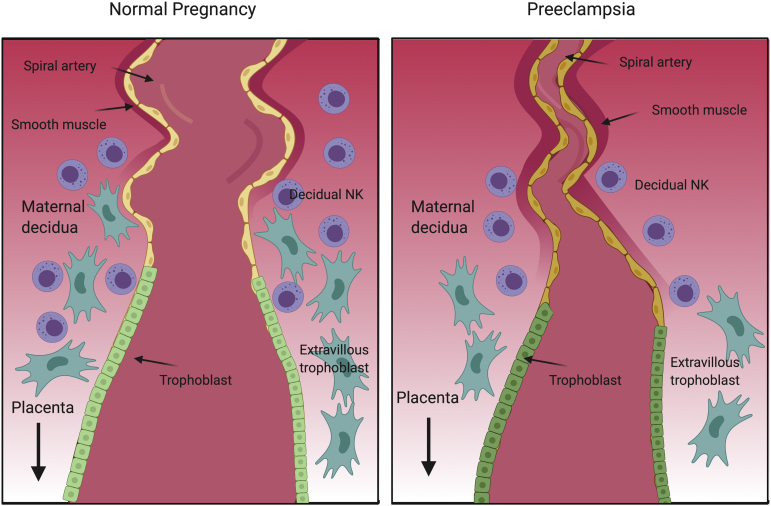
Spiral artery defects in PE In normal placental development, invasive cytotrophoblasts of fetal origin invade the maternal spiral arteries, transforming them from small-caliber resistance vessels to high-caliber capacitance vessels capable of providing placental perfusion that is adequate to sustain the growing fetus. During the process of vascular invasion, the cytotrophoblasts differentiate from an epithelial phenotype to an endothelial phenotype (*left panel*). In PE, cytotrophoblasts fail to adopt an invasive endothelial phenotype. Instead, the invasion of the spiral arteries is shallow, and they remain small-caliber, high-resistance vessels leading to placental ischemia (*right panel*). *NK*, natural killer; *PE*, preeclampsia. *Rana. Proangiogenic and antiangiogenic factors in preeclampsia. Am J Obstet Gynecol 2022.*

Human placentation is characterized by a deep invasion of the cytotrophoblasts into the maternal vasculature (Figure 1). In women destined to develop PE, the trophoblast invasion is shallow. This shallow implantation leads to placental hypoxia and stress, which in turn not only results in the release of circulating factors but also abnormal development of the placenta.^7^^,^^12^^,^^20^, ^21^, ^22^, ^23^, ^24^ Women with PE are shown to have defects in uterine artery flow, as evidenced by abnormal Doppler results in early pregnancy, suggesting that abnormal placentation precedes clinical disease onset.^25^ Gene expression studies on placentas obtained from women with PE mirror hypoxia-induced placental culture studies, thus providing molecular evidence that reducing oxygenation may contribute to the pathogenesis of PE.^26^ Placental hypoxia leads to aberrant expression and release of antiangiogenic factors that play a central role in the pathogenesis of the maternal syndrome.^27^, ^28^, ^29^, ^30^

The hypothesis that uteroplacental hypoperfusion leads to PE is supported by both animal and human studies.^22^^,^^28^^,^^31^, ^32^, ^33^ Placental ischemia induced by mechanical constriction of the uterine arteries or aorta produces hypertension, proteinuria, and glomerular endotheliosis in pregnant rats and baboons.^28^^,^^32^ There are extensive physiological changes and vascularization of the placental bed for the development of the fetus. In normal pregnancy, cytotrophoblast cells aggregate and anchor the embryo to the uterine wall. These extravillous cytotrophoblasts then invade the interstitium of the decidua and maternal uterine spiral arteries to provide the fetus a pathway for accessing nutrients and oxygen, while excreting waste products.^31^ To accomplish this, the placenta must form new blood vessels and increase the number of already existing blood vessels. This is facilitated by the release of angiogenic factors, such as vascular endothelial growth factor (VEGF) A and C isoforms, by decidual natural killer (dNK) cells and macrophages in the decidua.^34^, ^35^, ^36^ The uterine spiral arteries are transformed into low-resistance vessels by dilation and loss of elasticity even before the trophoblast invasion of the spiral arteries.^37^ The role of dNK cells has been extensively studied in animal models, and it is well accepted that dNK cells secrete cytokines and proangiogenic factors to support hemochorial placentation.^36^^,^^38^^,^^39^ Placental bed biopsy studies have reported that remodeling occurs in the spiral arteries as far as the inner third of the myometrium.^11^^,^^40^ During this process, extravillous trophoblast (EVT) cells change from an epithelial phenotype to a more endothelial phenotype (a process referred to as pseudovasculogenesis or vascular mimicry) as reflected by changes in the expression of cell surface adhesion molecules.^41^ The EVT cells widen and strengthen the diameter of the vessel walls, resulting in large-bore, low-resistance spiral arteries that can provide the growing fetus an optimal supply of blood. Histologic studies show that the physiological remodeling of the spiral arteries is incomplete in patients with PE.^24^^,^^42^ The spiral arteries in the myometrium retain their endothelial linings and muscular walls, thereby retaining their high-resistance, pulsatile phenotype. Defects in the phenotypic switching of cellular adhesion molecules on EVT, accompanied by a failure of vascular remodeling, may be the initial insult in the pathogenesis of PE.^43^ Although the etiology of this primary defect in PE is still unknown, defects in the natural killer (NK) cell function at the placental bed may at least provide an insight in some patients with preterm PE.^44^ It has been argued that dNK cells in the decidua interact with specific trophoblast cells from the fetus to mediate, and in some cases limit, trophoblast invasion of the decidua.^45^^,^^46^ This interaction takes place between the killer-cell immunoglobulin-like receptor (KIR) on the maternal NK cells and the human leukocyte antigen (HLA) receptor on the fetal trophoblast. Mothers carrying an inhibitory KIR (AA genotype) have been reported to be at an increased risk of PE when the fetus had a HLA-C2 allotype, a combination that is associated with impaired trophoblast invasion.^47^

Although defects in spiral artery remodeling and placental ischemia are predominantly seen in placentas from patients with PE, these events by themselves are not sufficient to induce PE, because many cases of idiopathic intrauterine growth restriction (IUGR) have similar placentation defects as noted in PE, but they do not develop the maternal syndrome. Therefore, it has been argued that a secondary insult to the syncytiotrophoblast layer of the placenta must occur after placental ischemia for the development of the maternal syndrome.^48^^,^^49^ Abnormalities in syncytialization and placental microparticle release have been reported in PE, but not in IUGR.^50^ Studies have reported that during PE, there is an increased release of placental syncytiotrophoblast extracellular vesicles, which can transfer placenta-specific proteins, messenger RNAs (mRNAs), and microRNAs to the maternal endothelium, thus inducing endothelial dysfunction by a paracrine fashion.^51^, ^52^, ^53^, ^54^ Thus, syncytiotrophoblast vesicles may cause endothelial damage and contribute to the endothelial dysfunction noted in PE.^55^ More studies are needed to understand the molecular apparatus that drives the formation of the syncytium and how placental ischemia may lead to the shedding of placental microparticles into the systemic circulation.

In addition to shallow trophoblast invasion, there is also a diffuse vascular injury observed in the decidual vessels away from any trophoblast invasion, which is referred to as decidual vasculopathy.^31^ Decidual vasculopathy can present as medial hypertrophy with perivascular lymphocytes or with fibrinoid necrosis within the vessel wall with occasional foam cells, which is termed as acute atherosis.^56^ Decidual vasculopathy is more commonly seen in the setting of PE complicated by IUGR.^56^, ^57^, ^58^ Impaired placentation with decreased uteroplacental blood flow is classically noted in early PE; however, similar changes (albeit to a milder degree) are also noted in term PE. Interestingly, placenta lesions and decreased uteroplacental flow are particularly prevalent among the term cases that have evidence of imbalance in circulating angiogenic factors.^59^

## Imbalance in Angiogenic Factors in Preeclampsia

**Figure 2:**
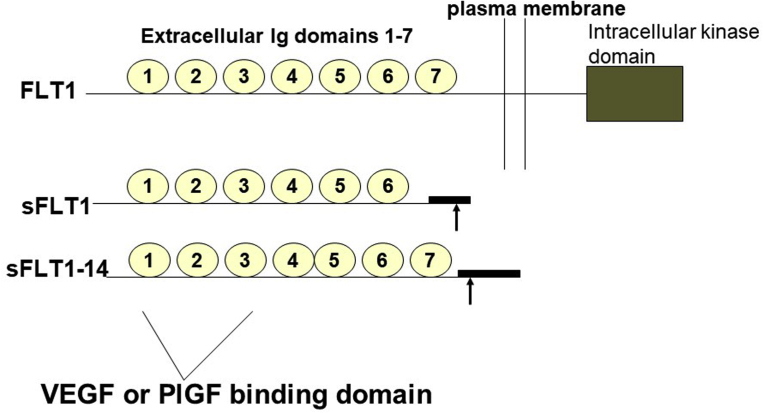
Schematic of the isoforms of placental sFLT1 sFLT1 belongs to a family of alternatively spliced proteins made by the placenta that lack the cytoplasmic and transmembrane domain of the full-length FLT1 receptor. Two major isoforms made in human placentas are shown as sFLT1 and sFLT1–14. sFLT1 family of proteins are secreted extracellularly into maternal circulation as they do not possess the anchoring domains that attach to cell membrane. *FLT1*, fms-like tyrosine kinase 1; *PlGF*, placental growth factor; *sFLT1*, soluble fms-like tyrosine kinase 1; *sFLT1–14*, soluble fms-like tyrosine kinase 1–14; *VEGF*, vascular endothelial growth factor. *Rana. Proangiogenic and antiangiogenic factors in preeclampsia. Am J Obstet Gynecol 2022.*

PE can be explained as a 2-stage process, where there is an initial preclinical phase early in pregnancy (first trimester) that is characterized by abnormal placentation and subsequently a symptomatic phase occurring later in pregnancy (>20 weeks’ gestation) that is characterized by the maternal syndrome of hypertension and multiorgan dysfunction.^60^ Many candidate molecules of placental origin have been hypothesized to explain the link between abnormal placentation and the maternal syndrome. Our group used transcriptional profiling to search for candidate factors produced by the placenta in PE. Using this approach, we found that soluble sFLT1 (or soluble VEGF receptor 1) mRNA is up-regulated in placentas obtained from women with PE.^13^ We then confirmed that circulating sFLT1 was markedly elevated in women with PE (n=21) compared with women with normotensive term deliveries (n=11) or women with normotensive preterm delivery (n=6).^13^ Many groups have subsequently confirmed that sFLT1 is dramatically overexpressed in preterm PE using either transcriptional profiling or RNA sequencing studies.^61^^,^^62^ sFLT1, a splice variant of the VEGF receptor, fms-like tyrosine kinase 1 (FLT1), which lacks the transmembrane and cytoplasmic domains, is made in large amounts by the placenta and is released into the maternal circulation.^14^^,^^63^ There are several variants of sFLT1, including the variant sFLT1–14, which is expressed only in primates^64^, ^65^, ^66^ (Figure 2). sFLT1 acts as a potent VEGF and placental growth factor (PlGF) antagonist by binding these molecules in the circulation. The expression of sFLT1 protein is largely localized to the syncytiotrophoblast layer and to abnormal clusters of degenerative syncytiotrophoblast, known as syncytial knots (also referred to as Tenney-Parker lesion).^64^

Circulating sFLT1 concentrations are increased in women with established PE^13^^,^^67^^,^^68^ and may begin to rise before the onset of clinical symptoms.^69^^,^^70^ Elevated sFLT1 is more pronounced in early-onset PE and PE complicated by IUGR.^71^^,^^72^ Consistent with the antagonistic effect of sFLT1, free (or unbound) VEGF and free (or unbound) PlGF concentrations are decreased in women with PE at the time of diagnosis and even before the onset of clinical symptoms.^13^^,^^69^^,^^73^^,^^74^ Indeed, when assays to measure total VEGF or total PlGF were employed, patients with PE were characterized by no reductions in the total levels of these ligands in the circulation.^75^^,^^76^ These data suggest that angiogenic imbalance noted in circulation during PE is largely driven by excess levels of sFLT1.

**Figure 3:**
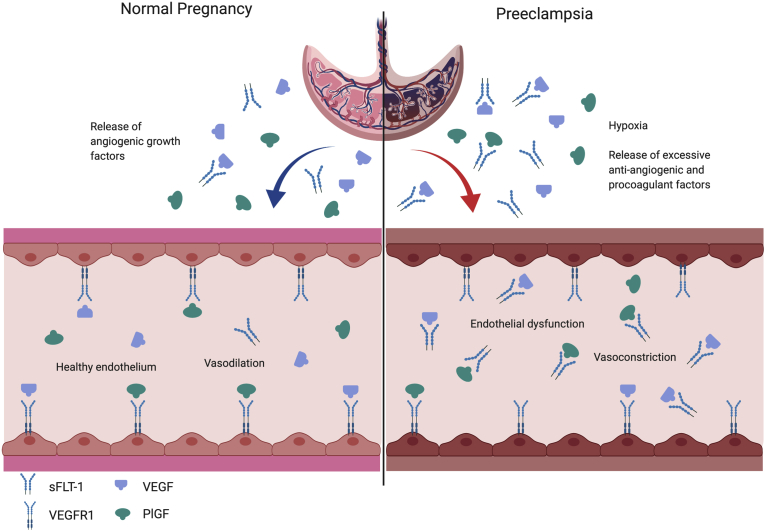
sFLT1 causes endothelial dysfunction by antagonizing VEGF and PlGF signaling VEGF and PlGF are necessary to maintain endothelial health in several tissues including the kidney and perhaps the placenta. During normal pregnancy, vascular homeostasis is maintained by physiological levels of VEGF and PlGF signaling in the vasculature. In PE, excess placental secretion of sFLT1 inhibits VEGF and PlGF signaling in the vasculature. This results in endothelial cell dysfunction, including decreased prostacyclins, nitric oxide production, and release of procoagulant proteins, leading to the clinical manifestations of PE. *PE*, preeclampsia; *PlGF*, placental growth factor; *sFLT1*, soluble fms-like tyrosine kinase 1; *VEGF*, vascular endothelial growth factor; *VEGFR1*, vascular endothelial growth factor receptor 1. *Rana. Proangiogenic and antiangiogenic factors in preeclampsia. Am J Obstet Gynecol 2022.*

In patients with PE, high circulating levels of sFLT1 contribute to maternal endothelial dysfunction and the clinical syndrome of PE.^71^ In vitro studies have reported that neutralizing antibodies against sFLT1 can reverse the antiangiogenic state in human preeclamptic plasma.^77^ sFLT1 impairs vascular endothelial health by binding to circulating VEGF and PlGF and inhibiting their mitogenic and homeostatic actions on endothelial cells.^63^^,^^77^ Pregnant rats administered with exogenous sFLT1 via adenoviral vector develop characteristic symptoms of PE including hypertension, proteinuria, and glomerular endotheliosis.^13^^,^^78^^,^^79^ Transgenic overexpression of sFLT1 overexpression in the mouse placenta or lentiviral transduction of sFLT1 into the mouse placenta was associated with reduced placental efficiency, enlarged maternal sinusoids, reduced fetal capillaries, and impaired labyrinthine differentiation, IUGR, and PE-like phenotype.^80^^,^^81^ Antagonism of sFLT1 led to an improvement in hypertension and renal phenotype in rodent models of PE with no adverse effects to the fetus.^82^, ^83^, ^84^ At least the hypertensive phenotype and endothelial dysfunction induced by sFLT1 may be caused by the interference of nitric oxide (NO) signaling and enhancing endogenous angiotensin II sensitivity^85^ (Figure 3). However, other factors such as increased endothelin secretion,^86^^,^^87^ prostacyclins, and hydrogen sulfide that are downstream of the VEGF receptor may also be involved.^88^^,^^89^

Up-regulated sFLT1 levels are also thought to contribute to the increased risk of PE in molar pregnancies,^90^^,^^91^ twin pregnancies,^92^ trisomy 13 pregnancies,^93^^,^^94^ women with antiphospholipid antibody syndrome,^95^ and women with preexisting diabetes mellitus.^96^ A large genome-wide association screening of >7 million genetic variants in 2658 offspring from women with PE and 308,292 population controls identified a single association signal in the FLT1 locus, on chromosome 13,^97^ providing support for an etiological role for the sFLT1 pathway in some patients with PE. These findings were confirmed recently in 2 independent Estonian cohorts.^98^

The use of VEGF inhibitors for the treatment of cancer-related angiogenesis in patients with cancer has been associated with hypertension, proteinuria, glomerular endothelial damage, elevated circulating liver enzymes, cerebral edema, and reversible posterior leukoencephalopathy—features resembling those found in human PE and eclampsia.^100^, ^101^, ^99^ These studies point to the central role of sFLT1 and impaired VEGF signaling in the development of PE. Several pathways have been proposed to induce PE, including mitochondrial stress, angiotensin II signaling, aberrant corin expression, decreased hemoxygenese expression, and deficient catechol-O-methyltransferase^102^, ^103^, ^104^, ^105^, ^106^, ^107^; however, no consensus has been reached on the role of these pathways in regulating sFLT1 in humans with PE.^108^

Although sFLT1 plays an important role in the pathogenesis of PE, it is unlikely that sFLT1 levels alone govern the disease onset. Endoglin (CD105) is a cell surface coreceptor for the transforming growth factor (TGF) family members such as TGF-β1 and TGF-β3. These 2 growth factors are potent inhibitors of trophoblast differentiation and migration.^109^ sENG is a truncated form of the extracellular domain of endoglin that is expressed at high levels by the syncytiotrophoblast layer and by cytotrophoblast cells undergoing differentiation to an invasive phenotype. Similar to sFLT1, circulating sENG levels are elevated weeks before PE onset.^110^ Endoglin is a cell surface receptor that binds to and regulates signaling of TGF-β.^111^ Recent studies have suggested that the antiangiogenic effects of sENG may be mediated by antagonizing the effects of bone morphogenetic protein 9, an antiangiogenic protein that controls vascular quiescence in the adult vasculature.^112^ Animals treated with both sFLT1 and sENG display severe signs of PE including hemolysis, elevated liver enzymes, and low platelet count (HELLP) syndrome; hemolysis; and thrombocytopenia.^16^^,^^113^ The effects of sENG can be mediated by interference with NO-mediated vasodilation, suggesting that NO production is downstream of sENG.^114^^,^^115^ In humans, sENG has been found to be particularly dramatically elevated in patients with severe complications of PE such as placental abruption, HELLP syndrome, eclampsia, and IUGR.^71^^,^^110^^,^^116^, ^117^, ^118^, ^119^ There is also some evidence that genetic polymorphism in the endoglin pathway may enhance susceptibility to PE. In a case-control study, it was reported that white women carrying an endoglin gene polymorphism were 2.29 times more likely to develop PE than women carrying wild-type genotypes. For black women, a similar evaluation of TGF-β1 polymorphisms revealed 7.44 times excess risk of PE in women carrying the polymorphism than those carrying the wild-type genotype.^120^ These data provide additional support for a role of endoglin pathway in the pathogenesis of PE.

## Clinical Implications

### Angiogenic biomarkers in the diagnosis and prognosis of preeclampsia

With the availability of commercial assays to reliably measure sFLT1 and PlGF, several studies have now confirmed that plasma or serum measurements of sFLT1 and PlGF when used alone or in combination have sensitivities and specificities of approximately 90% as an aid in diagnosis and prognosis of preterm PE.^79^^,^^121^, ^122^, ^123^, ^124^, ^125^, ^126^, ^127^, ^128^ The use of traditional clinical criteria for the diagnosis and management of PE has been shown to have poor predictive value for the detection of PE-related adverse outcomes.^129^ The use of clinical criteria is further complicated when women have preexisting signs and symptoms that overlap with clinical signs of PE, such as women with chronic hypertension and renal disease.^130^ Clinical management is challenging in these patients because overdiagnosis of PE can lead to iatrogenic prematurity and underdiagnosis can lead to adverse maternal outcomes. Given the nonspecific nature of clinical signs and symptoms necessary for the diagnosis of PE, it is important to develop tests that are specific to PE such that pregnancy can perhaps be continued in patients with unclear diagnosis and with normal angiogenic profiles.

Chaiworapongsa et al^131^ reported that in the suspected population with PE, although 1 in 10 pregnant women develops some signs and symptoms observed in PE, only approximately 20% of such patients are eventually diagnosed as having PE. Several prospective studies have been conducted to evaluate the plasma or serum levels of sFLT1-to-PlGF ratio among women with suspected PE, and sFLT1-to-PlGF ratio of <38 has shown a very high negative predictive value (NPV) for the short-term prediction of PE.^132^, ^133^, ^134^, ^135^ Moreover, angiogenic factors have been shown to aid in predicting short-term adverse outcomes including preterm delivery.^127^^,^^136^, ^137^, ^138^ In a large prospective study, we demonstrated that plasma sFLT1-to-PlGF ratio among women with suspected PE predicts adverse maternal and perinatal outcomes (occurring within 2 weeks) in the preterm setting (positive likelihood ratio, 12.2).^136^ This ratio alone outperformed standard clinical diagnostic measures including blood pressure, proteinuria, and uric acid. Importantly, sFLT1-to-PlGF ratios in the triage setting correlated with preterm delivery, an observation that has now been confirmed by several groups.^79^^,^^139^^,^^140^ In a similar but recent large prospective multisite observational study enrolling approximately 1000 women with suspected PE, sFLT1-to-PlGF ratio of <38 showed an NPV of 99.3% for the development of PE within 1 week (prevalence of outcome, 17.8%).^132^ Another study evaluating levels of PlGF for the time to delivery (n=753) found that among women with signs or symptoms of PE, women with PlGF of ≤100 pg/mL have a hazard ratio of 7.17 in Cox regression for the time to delivery compared with women with normal PlGF.^137^ In addition, studies have reported that women diagnosed as having PE, but with a normal plasma angiogenic profile, are not at risk of adverse maternal or fetal outcomes.^141^ In addition, several groups have demonstrated that sFLT1 and PlGF levels can be used to differentiate PE from diseases that mimic PE such as chronic hypertension, gestational hypertension, kidney disease, and gestational thrombocytopenia.^117^^,^^128^^,^^142^, ^143^, ^144^, ^145^ These observations have led to the incorporation of angiogenic factors for the prediction of PE in countries where these assays are available such as the National Institute for Health and Care Excellence (NICE), United Kingdom, and many parts of Europe.^146^

In a study in Haiti, we measured the plasma levels of sFLT1 and PlGF among women with PE and found that women with adverse outcomes (placental abruption, respiratory complications, stroke, renal insufficiency, eclampsia, maternal death, birthweight of <2500 g, or fetal or neonatal death) had a markedly elevated sFLT1-to-PlGF ratio regardless of the gestational age of presentation.^147^ Similar studies that have been conducted in other regions such as South Africa showed that black women with PE have significantly lower levels of PlGF, higher sFLT1, and a higher sFLT1-to-PlGF ratio than black normotensive controls.^148^, ^149^, ^150^

Because most studies showing association between angiogenic factors and PE are observational, randomized trials are now being done to determine whether information about plasma angiogenic factors in real time will improve patient outcomes. A recent multicenter, pragmatic, stepped-wedge cluster randomized controlled trial enrolling approximately 1000 women with suspected PE evaluated the effect of knowledge of plasma PlGF on the clinician’s decision making. The median time to PE diagnosis was 4.1 days with concealed testing vs 1.9 days with revealed testing (time ratio, 0.36; 95% confidence interval [CI], 0.15–0.7; *P*=.027). There was a significant improvement in maternal severe adverse outcomes, with no differences in perinatal outcomes or gestational age at delivery.^151^ Another prospective, randomized clinical trial evaluated the knowledge of serum sFLT1-to-PlGF ratio on hospitalization within 24 hours of the test among women with suspected PE (N=370). The reveal trial arm admitted 100% of the cases that developed PE within 7 days, whereas the nonreveal trial arm admitted 83% (*P*=.038). The use of the test yielded a sensitivity of 100% (95% CI, 85.8–100) and an NPV of 100% (95% CI, 97.1–100) compared with a sensitivity of 83.3 (95% CI, 58.6–96.4) and NPV of 97.8 (95% CI, 93.7–99.5) with clinical practice alone.^152^ We used preestablished biomarker cutoffs (38 and 85) among patients who were evaluated for PE and hypothetically assigned them to be admitted if they had a high ratio or discharged if they had a low ratio.^153^ We demonstrated that using biomarkers in addition to clinical decision may result in the reduction in admissions and increased prevalence of severe PE among those who are hospitalized. Randomized trials in the United States are needed to determine the effectiveness of angiogenic biomarker use in decision making in a triage setting among women with suspected PE. Furthermore, angiogenic factor thresholds for prospective studies should incorporate the matrix (serum vs plasma) before interpretation of the results, because significant differences have been reported for these factors in different matrices.^154^

### Angiogenic biomarkers in the prediction of preeclampsia

Although angiogenic factors have been shown to be altered at the time of clinical disease, these factors are also altered well before the onset of clinical signs and symptoms.^69^, ^70^, ^71^^,^^110^^,^^155^ Elevated sFLT1 and depressed PlGF levels are more dramatically altered in preterm PE and in PE complicated by IUGR.^110^^,^^156^ Kusanovic et al^157^ measured sFLT1, PlGF, and sENG in 1622 consecutive singleton pregnant women during early pregnancy and in midtrimester and found superior performances for the PlGF-to-sENG ratio during midtrimester with a sensitivity of 100%, a specificity of 98%, and a positive likelihood ratio of 57.6 for predicting early-onset PE. Sovio et al^158^ reported that among a group of unselected nulliparous women, elevations of plasma sFLT1-to-PlGF ratio of >38 at 28 weeks’ gestation were associated with a positive likelihood ratio of 70.3 for the development of preterm PE.

Serum levels of PlGF tend to be lower in women who go on to develop PE from the first or early second trimester.^159^^,^^160^ Because PlGF alterations occur early in the first trimester, PlGF has been tested alone and in combination with other biomarkers as a potential predictive test. In a large prospective clinical study involving approximately 8000 subjects, Poon et al^161^ demonstrated that a combination of PlGF, pregnancy-associated plasma protein A, and uterine artery Doppler velocimetry in the first trimester can predict the subsequent development of early-onset PE in a low-risk population with a sensitivity of 93% at a 5% false-positive rate. This suggests that screening for early-onset PE in the general population is possible and that 1 in 5 pregnancies that had a positive screening result would develop PE. Current evidence suggests that screening strategies proposed by the American College of Obstetricians and Gynecologists and NICE that rely entirely on clinical characteristics are suboptimal compared with the first-trimester prediction model proposed by Poon et al^161^ at the Fetal Medicine Foundation (FMF). The use of the FMF algorithm followed by the administration of low-dose acetylsalicylic acid (ASA, aspirin) has been associated with a 62% reduction in preterm PE.^162^

### Urine biomarkers

Because PlGF is freely filtered by the glomerulus into the urine, the measurement of urinary PlGF has emerged as attractive biological fluid to screen for PE. Urine PlGF measured in midpregnancy was first reported to be associated with the subsequent development of preterm PE.^163^ Follow-up studies by other groups have confirmed that urine PlGF is a robust marker of PE.^164^ For a fixed false-positive rate of 10%, the sensitivity of urinary PlGF concentrations at midpregnancy was 75% for the subsequent development of PE-related adverse outcomes.^165^ Currently, urine PlGF is only used in the research context because the automated assays for PlGF have not yet been validated in pregnant urine samples.

### Cost-effectiveness of the use of biomarkers in preeclampsia

In United States, PE drives substantial short-term prenatal and postnatal care costs and more than $2 billion in costs at 1 year after delivery.^166^ Obviously, not all cost will be mitigated by the use of biomarkers. The measurement of sFLT1 and PlGF is not yet approved in the United States. However, research studies conducted in the United States using a hypothetical scenario have shown cost savings with the use of sFLT1-to-PlGF ratio in triage resulting from the reduction in false-positive results compared with the current standard of care, that is, $1215 per patient.^167^ Economic models evaluating the incremental value of sFLT1-to-PlGF ratio in the United Kingdom is expected to result in cost savings of £344 per patient compared with a no-test scenario.^168^ Savings are generated primarily through an improvement in diagnostic accuracy and subsequent reduction in unnecessary hospitalization. Using data from the Placental Growth Factor to Assess and Diagnose Hypertensive Pregnant Women: A Stepped Wedge Trial, investigators reported that clinical care with PlGF tests would lead to cost savings in the United Kingdom of £2,891,196 per year.^169^ Prospective studies in various geographic areas incorporating the cost of PE management with biomarkers need to be performed.

### Implications for therapeutic studies

Human and animal studies as outlined earlier strongly suggest that targeting angiogenic factor imbalance and in particular sFLT1 may be a viable strategy to prevent or treat PE.^170^ Thadhani et al^171^ recently translated some fundamental discoveries to the bedside. Taking advantage of the positive charge of sFLT1, they used a negatively charged dextran sulfate cellulose column for the extracorporeal removal of sFLT1. In 2 studies including women with preterm PE, dextran sulfate apheresis led to a reduction in sFLT1 levels and improvement in proteinuria and blood pressure, without evident adverse effects to the mother and fetus. In all of these cases, there was evidence of continued fetal growth and prolongation of pregnancy.^171^^,^^172^ Other modalities for targeting the angiogenic imbalance are the administration of agents that scavenge sFLT1, such as sFLT1 antibodies, recombinant PlGF, or recombinant VEGF, and decreased sFLT1 production by small interfering RNA strategies or small molecules is currently being evaluated in the preclinical settings.^170^^,^^173^ Compounds that up-regulate proangiogenic factors such as statins have been demonstrated to reverse PE in animal models^81^ and have prolonged pregnancy in few cases of severe preterm PE.^174^^,^^175^ A clinical trial to test the safety of statins in women with established PE has been initiated in the United States.^176^ A small study demonstrated that higher maternal choline diet in normal pregnant women was associated with a lower circulating concentrations of sFLT1^177^; however, it is not known whether such an approach will prevent PE.

ASA is often used for the prevention of PE. The effect of ASA on angiogenic factors, such as sFLT1 and PlGF, is the subject of ongoing interest and has been examined by several groups. Mechanisms explored in vitro include the effect of low-dose ASA on cyclooxygenase pathway–induced sFLT1 expression,^178^ trophoblast cell integration into endothelial cellular networks by inhibiting the effect of TNF-α,^179^ or inhibition of sFLT1 production via the Akt pathway in cytotrophoblasts.^180^ In a recent study of 394 women at risk of the development of PE and who were prescribed with ASA, the authors found that the use of ASA showed a trend toward a reduction of the sFLT1-to-PlGF ratio in women with PE in a previous pregnancy and a significant effect on the sFLT1-to-PlGF ratio in women with a pathologic first-trimester screening for PE.^181^

## Role of Angiogenic Markers in Other Placentation Disorders

### Intrauterine growth restriction

Small for gestational age (SGA) is defined as fetal measurements at the <10th percentile for gestational age determined by a sonographic assessment of the fetal weight. At least some forms of SGA may be manifestations of placental insufficiency (referred to as IUGR), and angiogenic factors may allow us to classify placenta-mediated SGA (IUGR) vs nonpathologic etiologies of SGA (such as being constitutionally small). Many studies have reported that even in women without PE, angiogenic factors in maternal circulation are strongly associated with the delivery of an SGA infant.^156^ In particular, an angiogenic profile characterized by low sFLT1 in the first trimester, followed by a strong increase in sFLT1 relative to PlGF, combined with high levels of sENG in the second trimester, was associated with a very high SGA risk.^118^ Angiogenic factors may display similar patterns in relation to both PE and SGA, but nonetheless, the absolute angiogenic factor levels may differ. From some studies that included both SGA and PE, it may be suggested that sFLT1 levels are higher in women with PE than normotensive women with SGA delivery.^156^^,^^182^ Therefore, it is possible that very high maternal sFLT1 levels are required for PE to develop. Chaiworapongsa et al^183^ suggested that elevated midtrimester plasma angiogenic profile among patients carrying an SGA infant helped identify women who subsequently developed PE or who delivered preterm. Sharp et al^184^ recently reported that among patients with early-onset IUGR, overall survival can be predicted using a model of estimated fetal weight and sFLT1-to-PlGF ratio. Gaccioli et al^185^ confirmed that a high sFLT1-to-PlGF ratio in sonographically suspected SGA pregnancies in nulliparous women identified women with high absolute risks of adverse perinatal outcomes.

### Angiogenic factors in fetal death and massive perivillous fibrin deposition syndrome

Although angiogenic factors have mostly been studied in the context of PE, there is a large body of evidence that angiogenic profile is reflective of placental disease regardless of diagnosis of PE. Most importantly, angiogenic factor abnormalities have been identified among patients with fetal death unrelated to PE. In a study by Espinoza et al,^186^ plasma sFLT1 levels were higher among patients with fetal deaths than normal pregnant women, and plasma concentrations of sFLT1 and PlGF at 30 to 34 weeks’ gestation were useful for the risk assessment for stillbirth in the third trimester.^187^ In another study by Chaiworapongsa et al,^188^ amniotic fluid concentrations of sFLT1 and sENG were measured among patients with fetal death and controls. The authors found that patients with an unexplained fetal death were characterized by an increase in the amniotic fluid concentrations of sFLT1 and sENG. In a more recent large case-control study by the same group,^189^ the authors examined the angiogenic profile at 24 to 28 weeks’ gestation among patients with fetal death and controls. The authors showed that patients with a low plasma angiogenic index (PlGF to sFLT1), that is, <2.5th centile, carry a 29-fold increased subsequent fetal death and the plasma angiogenic index identified 55% of subsequent fetal deaths with a false-positive rate of 3.5%. Approximately 60% of women with a false-positive test result subsequently experienced adverse pregnancy outcomes. Another lesion of the placenta called massive perivillous fibrin deposition (MPFD) is associated with serious complications of pregnancy including recurrent spontaneous abortion, IUGR, and fetal demise. In a study involving patients with MPFD and controls, the authors show that patients with MPFD had a lower plasma PlGF concentration and higher mean plasma concentrations of sFLT1 and sENG, and the differences in these concentrations were observed before the diagnosis of MPFD, suggesting that antiangiogenic factors participate in the pathogenesis of these placental lesions.^190^

### Angiogenic factors in bronchopulmonary dysplasia

Angiogenic imbalance is also associated with short-term and long-term fetal complications such as bronchopulmonary dysplasia (BPD). Amniotic fluid levels of sFLT1 have been reported to be markedly elevated in patients with PE.^191^ In animals, excess sFLT1 in amniotic fluid is associated with BPD often seen in infants born prematurely.^192^ Several, but not all, epidemiologic studies report an association with excess BPD in infants born to preeclamptic mothers.^193^ PE may leave a persistent defect in the pulmonary circulation of the offspring. This defect predisposes to exaggerated hypoxic pulmonary hypertension already during childhood and may contribute to premature cardiovascular disease (CVD) seen later in life in offsprings of mothers with PE.^194^

### Angiogenic factors in fetal hydrops, Ballantyne syndrome, and twin-to-twin transfusion syndrome

Fetal hydrops is often associated with maternal hypertension and PE-like syndrome that is referred to as mirror syndrome or Ballantyne syndrome.^195^^,^^196^ In a case report, we described a case of maternal PE associated with fetal hydrops with high levels of sFLT1 and sENG and low levels of PlGF. The placental staining confirmed the source of sFLT1 and sENG to be placental in origin.^197^ Other reports have described similar high levels of sFLT1 and low levels of PlGF with fetal hydrops secondary to a variety of etiologies such as parvovirus infection,^198^ cytomegalovirus infection,^199^ and fetal heart failure,^200^ suggesting that the antiangiogenic state constitutes a link between fetal-placental unit and the clinical manifestation of PE in mirror syndrome. Interestingly, in the case of fetal hydrops secondary to parvovirus, treatment of fetal anemia with transfusion leads to a reduction of circulating maternal sFLT1 and consequent resolution of maternal syndrome.^198^^,^^201^ These reports not only demonstrate an association between enlargement of the placenta in the setting of fetal anemia or heart failure, extremely high maternal plasma levels of sFLT1, and signs of PE but also suggest that elevated levels of sFLT1 may be triggered by various forms of placental pathology. These reports also support the notion that interventions to reduce sFLT1 levels can be a potential avenue for the treatment of PE.

Another condition of placental dysfunction associated with angiogenic imbalance is twin-to-twin transfusion syndrome (TTTS). TTTS is characterized by abnormal vascular anastomosis and affects approximately 10% to 15% of the monochorionic twin pregnancies.^202^ Patients with TTTS have been shown to have higher levels of sFLT1 and sENG and low levels of PlGF compared with uncomplicated monochorionic diamniotic twin pregnancies,^203^ suggesting that TTTS is an antiangiogenic state.^204^^,^^205^ It is unclear whether an abnormal angiogenic profile contributes to the development of TTTS or whether an antiangiogenic state is a consequence of TTTS. In a recent study, laser correction of TTTS resulted in the reduction of sFLT1 levels,^206^ suggesting that perhaps antiangiogenic profile is a consequence of TTTS. Further studies are needed to evaluate whether antiangiogenic levels can predict the development of TTTS and guide the management and results of successful laser therapy.

## Angiogenic Factors and Cardiovascular Disease

Circulating angiogenic factors measured during the third trimester have also been implicated in immediate postpartum hypertension and peripartum morbidity.^207^^,^^208^ Studies using advanced echocardiography have also shown that global longitudinal strain (GLS) with speckle tracking echocardiography is a sensitive measure of the onset of subclinical cardiac dysfunction in PE, and these changes correlate with alterations in circulating sFLT1 and sENG.^209^^,^^210^ Because GLS differentiates active from passive contraction and is less sensitive to loading conditions than ejection fraction, it reliably detects the early onset of subclinical systolic heart failure in patients with PE.^195^^,^^209^^,^^211^^,^^212^

PE is also identified as a major risk factor for peripartum cardiomyopathy.^213^^,^^214^ In patients with preserved ejection fraction, GLS is sensitive to detect early subclinical cardiomyopathy before a decline in ejection fraction.^215^ Using animal models and human studies in women with peripartum cardiomyopathy, excess sFLT1 seen in late pregnancy has been reported to be a key link between the excess risk of cardiomyopathy and PE.^216^

Although PE has previously been considered only a peripartum disease, it is now recognized as a prominent risk factor for the long-term development of CVD. Long-term epidemiologic studies have shown an increased risk of chronic hypertension, premature CVD, and death in women with a history of PE.^217^, ^218^, ^219^, ^220^ Some have hypothesized that the underlying metabolic milieu of these women (shared risk factors) confers risk of both PE and long-term CVD. A large Norwegian study suggested that at least 50% of the long-term hypertension can be explained by preexisting risk factors.^221^ However, it is also possible that subtle vascular injury induced by PE, subclinical in nature after the resolution of proteinuria and hypertension, leads to chronic hypertension, premature atherosclerosis, and death. The absence of hypertension in the siblings of women with PE who might be expected to be at a similar risk of CVD^222^ and an increased risk of CVD in women with recurrent PE supports the latter hypothesis.^223^ In experimental studies, exposure to PE in a mouse model induces angiotensin II sensitivity and exacerbates the vascular proliferative and fibrotic responses to future vascular injury.^224^ These data suggest that enhanced vascular remodeling response may contribute to the increased risk of future hypertension and CVD in women with a history of PE. Consistent with this hypothesis, in a large prospective cohort study of 5475 women, women with midtrimester decreases in PlGF (a marker of angiogenic imbalance) were associated with larger left ventricular mass and higher average systolic blood pressure 6 to 9 years after pregnancy than women with higher PlGF.^225^

## Conclusions

PE and related disorders have a large burden of disease worldwide and are a leading cause of maternal and infant morbidity. In areas where access to prenatal care is limited, patients present late with advanced disease and experience severe complications. The search for the ability to better diagnose, predict, and prevent PE and its related complications and the mechanisms of its pathogenesis to develop a therapy that safely prolongs gestation has been extensive. Exciting data on angiogenic factors as central contributors to the pathogenesis of PE, candidate biomarkers, and therapeutic targets have opened clinically meaningful options for patients now and in the immediate future. Recent evidence suggests that angiogenic biomarkers may also serve as surrogates of placental function and thus serve as clinically useful biomarkers for other placentation disorders such as idiopathic IUGR and intrauterine fetal death. PE and pregnancies complicated by preterm delivery are associated with excess CVD in the long-term. Understanding the mechanisms by which angiogenic factors lead to persistent endothelial dysfunction and acceleration of CVD has potential to advance the prevention of heart disease in this large and expanding population of high-risk women.

**Glossary of Terms**

**ASA:** acetylsalicylic acid.

**Angiogenesis:** A physiological process where new blood vessels grow from preexisting blood vessels. Factors that regulate angiogenesis are referred to as “angiogenic factors.” A factor such as vascular endothelial growth factor that promotes angiogenesis is referred to as a proangiogenic factor and a factor such as soluble fms-like tyrosine kinase 1 that blocks angiogenesis is referred to as an antiangiogenic factor.

**Apheresis:** Extracorporeal methods to remove toxic factors from the blood. Apheresis is most often used to treat autoimmune disorders to remove pathogenic autoantibodies.

**Atherosis:** The morphologic lesions of decidual vasculopathy can include fibrinoid necrosis and foam cell incorporation within the vessel wall resembling early forms of atherosclerosis and are therefore sometimes referred to as acute atherosis.

**BPD:** bronchopulmonary dysplasia.

**CI:** confidence interval.

**CVD:** cardiovascular disease.

**dNK:** decidual natural killer.

**Decidual vasculopathy:** This is a pathologic lesion often noted in women in preeclampsia that is diagnosed when perivascular lymphocytic infiltration, fibrinoid necrosis, and foam cell incorporation are noted within arterioles in the placental membranes away from trophoblast invasion.

**Endothelial dysfunction:** Endothelial damage leading to an impaired function of the endothelium (such as impaired vasodilation or procoagulant state) is referred to as endothelial dysfunction.

**EVT:** extravillous trophoblast.

**Fetal hydrops:** This is a serious fetal condition defined as an abnormal accumulation of fluid in ≥2 fetal compartments, including ascites, pleural effusion, pericardial effusion, and skin edema. In some patients, it may also be associated with polyhydramnios and placental edema.

**FLT1:** fms-like tyrosine kinase 1.

**FMF:** Fetal Medicine Foundation.

**Glomerular endotheliosis:** Glomerular endotheliosis is a classic histologic abnormality noted in the kidneys of women with preeclampsia.^226^ The lesion is a type of thrombotic microangiopathy of the kidney that is characterized by glomerular endothelial swelling with a loss of endothelial fenestrae and occlusion of the capillary lumens. When glomerular endotheliosis is present in a diffuse manner, in the appropriate clinical setting, it is virtually pathognomonic for preeclampsia.

**GLS:** global longitudinal strain.

**HELLP:** hemolysis, elevated liver enzymes, and low platelet count syndrome.

**HLA:** human leukocyte antigen.

**IUGR:** intrauterine growth restriction.

**KIR:** killer-cell immunoglobulin-like receptor.

**Microangiopathy:** Microangiopathy refers to disorders that predominantly affect small blood vessels in the body. Preeclampsia is a microangiopathic disease because the damaged vessels (eg, decidual vasculopathy affects arterioles in placental membranes) are mostly small vessels or capillaries in the body.

**MPED:** massive perivillous fibrin deposition.

**mRNA:** messenger RNA.

**NICE:** National Institute for Health and Care Excellence.

**NK:** natural killer.

**NO:** nitric oxide.

**NPV:** negative predictive value.

**PE:** preeclampsia.

**PlGF:** placental growth factor.

**sENG:** soluble endoglin.

**sFLT1:** soluble fms-like tyrosine kinase 1.

**SGA:** small for gestational age.

**Spiral artery:** Spiral arteries, so called because of their coiled appearance, supply blood to the endometrial layer and, in the pregnant uterus, span the inner myometrium and the decidua. During pregnancy, the placental bed spiral arteries are transformed from high-resistance, low-flow vessels into large dilated vessels with an increased blood flow at a much reduced pressure.^227^

**Syncytial knots:** Syncytial knots are aggregates of syncytial nuclei at the surface of terminal villi in the placenta. Syncytial knots are consistently present, increasing with increasing gestational age, and can be used to evaluate villous maturity. Increased syncytial knots are often noted with conditions of uteroplacental malperfusion such as preeclampsia or severe intrauterine growth restriction.

**TGF:** transforming growth factor.

**TTTS:** twin-to-twin transfusion syndrome.

**VEGF:** vascular endothelial growth factor.

**Vasculogenesis:** A physiological process where new blood vessels form de novo when there are no preexisting blood vessels. During vasculogenesis, endothelial precursor cells, referred to as angioblasts, migrate and differentiate in response to local cues (such as growth factors and extracellular matrices) to form new blood vessels.
